# Impact of left atrial appendage flow velocity on thrombus resolution and clinical outcomes in patients with atrial fibrillation and silent left atrial thrombi: insights from the LAT study

**DOI:** 10.1093/europace/euae120

**Published:** 2024-05-01

**Authors:** Masato Okada, Koichi Inoue, Nobuaki Tanaka, Koji Tanaka, Yuko Hirao, Katsuomi Iwakura, Yasuyuki Egami, Masaharu Masuda, Tetsuya Watanabe, Hitoshi Minamiguchi, Takafumi Oka, Shungo Hikoso, Akihiro Sunaga, Katsuki Okada, Daisaku Nakatani, Yohei Sotomi, Yasushi Sakata, Osaka Cardiovascular Conference (OCVC)-Arrhythmia Investigators, Masaharu Masuda, Toshiaki Mano, Koichi Inoue, Yasushi Matsumura, Masato Kawasaki, Tetsuya Watanabe, Takahisa Yamada, Miwa Miyoshi, Takashi Kanda, Hitoshi Minamiguchi, Nobuhiko Makino, Yoshiharu Higuchi, Yasuharu Matsunaga, Yasuyuki Egami, Masami Nishino, Jun Tanouchi, Taiki Sato, Hirota Kida, Akihiro Sunaga, Tomoaki Nakano, Kentaro Ozu, Yohei Sotomi, Tomoharu Dohi, Katsuki Okada, Takafumi Oka, Toshihiro Takeda, Daisaku Nakatani, Shungo Hikoso, Yasushi Sakata, Nobuaki Tanaka, Koji Tanaka, Masato Okada, Tomoko Minamisaka, Shiro Hoshida

**Affiliations:** Cardiovascular Centre, Sakurabashi Watanabe Hospital, 2-4-32 Umeda, Kita-ku, Osaka 530-0001, Japan; Cardiovascular Centre, Sakurabashi Watanabe Hospital, 2-4-32 Umeda, Kita-ku, Osaka 530-0001, Japan; Cardiovascular Division, National Hospital Organization Osaka National Hospital, Osaka, Japan; Cardiovascular Centre, Sakurabashi Watanabe Hospital, 2-4-32 Umeda, Kita-ku, Osaka 530-0001, Japan; Cardiovascular Centre, Sakurabashi Watanabe Hospital, 2-4-32 Umeda, Kita-ku, Osaka 530-0001, Japan; Cardiovascular Centre, Sakurabashi Watanabe Hospital, 2-4-32 Umeda, Kita-ku, Osaka 530-0001, Japan; Cardiovascular Centre, Sakurabashi Watanabe Hospital, 2-4-32 Umeda, Kita-ku, Osaka 530-0001, Japan; Division of Cardiology, Osaka Rosai Hospital, Osaka, Japan; Cardiovascular Centre, Kansai Rosai Hospital, Amagasaki, Japan; Division of Cardiology, Osaka General Medical Centre, Osaka, Japan; Cardiovascular Division, Osaka Police Hospital, Osaka, Japan; Department of Cardiovascular Medicine, Osaka University Graduate School of Medicine, Suita, Japan; Department of Cardiovascular Medicine, Osaka University Graduate School of Medicine, Suita, Japan; Department of Cardiovascular Medicine, Nara Medical University, Nara, Japan; Department of Cardiovascular Medicine, Osaka University Graduate School of Medicine, Suita, Japan; Department of Cardiovascular Medicine, Osaka University Graduate School of Medicine, Suita, Japan; Department of Medical Informatics, Osaka University Graduate School of Medicine, Osaka, Japan; Department of Cardiovascular Medicine, Osaka University Graduate School of Medicine, Suita, Japan; Department of Cardiovascular Medicine, Osaka University Graduate School of Medicine, Suita, Japan; Department of Cardiovascular Medicine, Osaka University Graduate School of Medicine, Suita, Japan

**Keywords:** Atrial fibrillation, Left atrial appendage flow velocity, Left atrial thrombus, Oral anticoagulant, Transesophageal echocardiography

## Abstract

**Aims:**

Blood stasis is crucial in developing left atrial (LA) thrombi. LA appendage peak flow velocity (LAAFV) is a quantitative parameter for estimating thromboembolic risk. However, its impact on LA thrombus resolution and clinical outcomes remains unclear.

**Methods and results:**

The LAT study was a multicentre observational study investigating patients with atrial fibrillation (AF) and silent LA thrombi detected by transoesophageal echocardiography (TEE). Among 17 436 TEE procedures for patients with AF, 297 patients (1.7%) had silent LA thrombi. Excluding patients without follow-up examinations, we enrolled 169 whose baseline LAAFV was available. Oral anticoagulation use increased from 85.7% at baseline to 97.0% at the final follow-up (*P* < 0.001). During 1 year, LA thrombus resolution was confirmed in 130 (76.9%) patients within 76 (34–138) days. Conversely, 26 had residual LA thrombi, 8 had thromboembolisms, and 5 required surgical removal. These patients with failed thrombus resolution had lower baseline LAAFV than those with successful resolution (18.0 [15.8–22.0] vs. 22.2 [17.0–35.0], *P* = 0.003). Despite limited predictive power (area under the curve, 0.659; *P* = 0.001), LAAFV ≤ 20.0 cm/s (best cut-off) significantly predicted failed LA thrombus resolution, even after adjusting for potential confounders (odds ratio, 2.72; 95% confidence interval, 1.22–6.09; *P* = 0.015). The incidence of adverse outcomes including ischaemic stroke/systemic embolism, major bleeding, or all-cause death was significantly higher in patients with reduced LAAFV than in those with preserved LAAFV (28.4% vs. 11.6%, log-rank *P* = 0.005).

**Conclusion:**

Failed LA thrombus resolution was not rare in patients with AF and silent LA thrombi. Reduced LAAFV was associated with failed LA thrombus resolution and adverse clinical outcomes.

## Graphical Abstract

**Graphical abstract:**
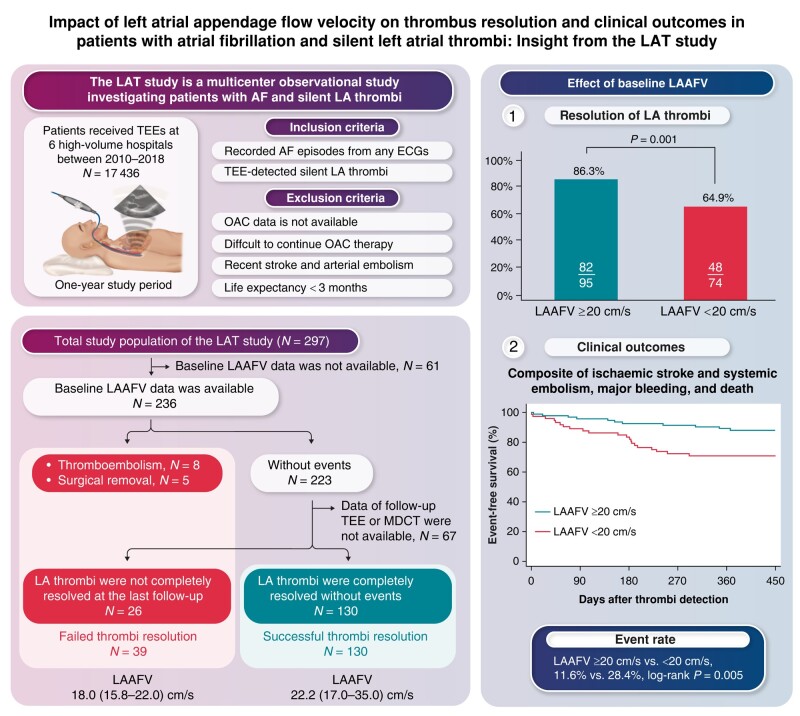

**What’s new?**

- The resolution of left atrial (LA) thrombi was evaluated in patients with atrial fibrillation (AF) by repeat transoesophageal echocardiography and/or multidetector computed tomography. Most patients achieved successful thrombus resolution; however, unsuccessful cases were not rare.
- Reduced left atrial appendage flow velocity (LAAFV) at baseline (cut-off value of <20.0 cm/s in this study) was associated with unsuccessful LA thrombus resolution, thromboembolism, or surgical removal.
- Reduced LAAFV was associated with adverse clinical outcomes in patients with AF and silent LA thrombi. Careful clinical follow-up and continuation of oral anticoagulants may be necessary to prevent adverse outcomes in patients with reduced LAAFV.

## Introduction

Atrial fibrillation (AF) is the most common cardiac arrhythmia and a well-established risk factor for thromboembolic complications.^1^ AF induces blood stasis, hypercoagulability state, and prothrombotic endothelial changes.^2,3^ These factors are crucial in left atrial (LA) thrombi formation, with the LA appendage (LAA) being the primary site.^4–8^

Oral anticoagulant (OAC) therapy has effectively decreased the prevalence of LA thrombi.^4,5^ Nonetheless, a small proportion of patients still develop LA thrombi despite continuous OAC therapy (up to 3% in the recent systematic review and meta-analysis).^4,5^ Additionally, with the emergence of direct oral anticoagulants (DOACs), management of persistent LA thrombi refractory to conventional OACs is increasingly important.^6–9^ Early identification of such patients would be useful for tailoring subsequent treatment approaches.

The LAA peak flow velocity (LAAFV), assessed by transoesophageal echocardiography (TEE), is an established quantitative parameter for estimating LA thrombi and thromboembolic events.^4,5,9–12^ A reduced LAAFV reflects blood slowing or pooling within the LAA. An increased prevalence of LA thrombi in patients with reduced LAAFV illustrates how ‘blood stasis’ is an important contributor to the development of LA thrombi. Conversely, it remains unknown whether ‘blood stasis’ also contributes to LA thrombus resolution. In this *post hoc* analysis of the LAT study, we aimed to investigate whether the LAAFV could be a useful parameter to predict failed LA thrombus resolution during an OAC therapy.

## Methods

### Study population

The LAT study is a retrospective, multicentre, observational study conducted by the Osaka Cardiovascular Conference (OCVC) with the participation of members from six high-volume hospitals that comprise the OCVC-arrhythmia team. The OCVC organization and data collection methods of this registry have been reported elsewhere.^13,14^ Briefly, the LAT study investigated the outcomes of silent LA thrombi detected by TEE in AF patients. The primary inclusion criteria were as follows: (1) recorded AF episodes from any electrogram (ECG) (e.g. 12-lead ECG, Holter recordings, monitor ECG, and so on) and (2) the presence of LA thrombi detected by TEE. The exclusion criteria were as follows: (1) data regarding the use of OACs were not available, (2) continuation of OAC treatment was difficult, (3) recent stroke and/or arterial embolism (<1 month), and (4) life expectancy of <3 months.

**Figure 1:**
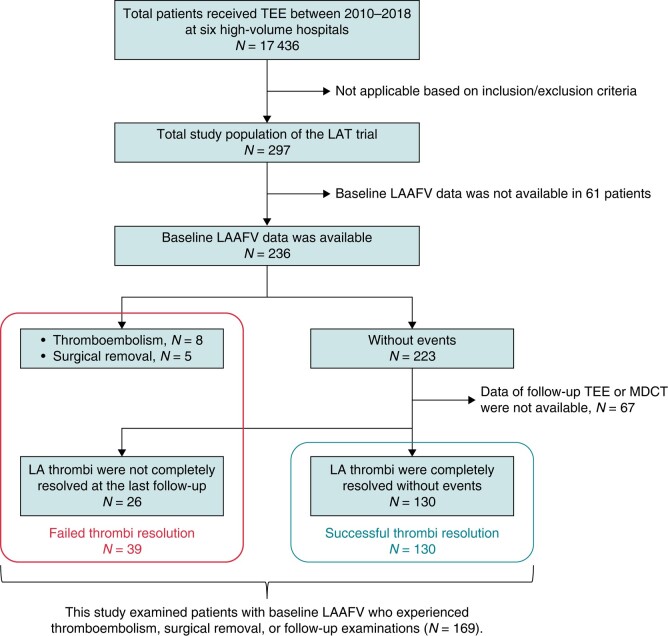
Study flow chart. LA, left atrial; LAAFV, left atrial appendage peak flow velocity; TEE, transoesophageal echocardiography; MDCT, multidetector computed tomography.

Initially, the trial was scheduled to enrol 100 patients with silent LA thrombi between January 2010 and December 2012. However, given the widespread use of DOACs^15^ and the development of various interventional techniques for LAA (e.g. transcatheter percutaneous closure and endoscopy-guided LA resection),^16^ the study protocol was revised, and the study period was extended. In the present analysis of the LAT study, among 17 436 AF patients who underwent TEE between January 2010 and March 2018, 297 (1.7%) (AF patients with silent LA thrombi identified by TEE) were eligible for inclusion. Among them, baseline LAAFV was available for 236 patients. During the 1-year study period, eight patients experienced thromboembolisms (i.e. four cardiogenic strokes, one atherothrombotic stroke, and three with acute limb ischaemia) (see Supplementary material online, *Table S1*), and five experienced surgical removal (see Supplementary material online, *Table S2*). No patients underwent percutaneous LAA closure. Of the 223 without these events, 156 patients underwent follow-up TEE or multidetector computed tomography (MDCT). Then, we enrolled 169 patients with baseline LAAFV who had thromboembolism, surgical removal, or follow-up examination in this study (*Figure 1*).

All data were retrospectively collected from the patients’ medical records. The investigators determined thrombus identification and resolution from TEE or MDCT imaging without blinded adjudication. Owing to the study’s retrospective design, written informed consent was not obtained; instead, the opt-out method was used. The present study was approved by the Institutional Review Board of each participating hospital and followed the ethical guidelines outlined in the Declaration of Helsinki.

### Transesophageal echocardiography

Transoesophageal echocardiography was performed using commercially available ultrasound systems owned by each hospital. The objective of TEE was thrombus check before cardioversion or catheter ablation (*n* = 166), vegetation check in patients with suspected infective endocarditis (*n* = 2), and preoperative valve evaluation (*n* = 1). After administering local pharyngeal anaesthesia with lidocaine spray, the patient was placed in the left lateral position, and the transoesophageal transducer was inserted into the oesophagus. The LAA images were observed in at least four directions (0°, 45°, 90°, and 135°) to evaluate the presence or absence of a thrombus, spontaneous echo contrast, patent foramen ovale, and the LAAFV. LA thrombi were defined as discrete echo-dense masses in the left atrium or LAA with different echo densities from the adjacent endocardium and independent motion relative to the chamber wall. When the LA thrombi were identified, the number of thrombi, location (LAA thrombi or LA thrombi outside the LAA), size (maximum length of the long and short axes), and morphology (pedunculated or floating) were evaluated. Then, the LAA outflow (emptying) and inflow (filling) velocities were obtained by pulsed-wave Doppler interrogation at the LAA entry (see Supplementary material online, *Figure S1*). The velocities were determined during 10 cardiac cycles, and the average value was used for representative measurements. When both the inflow and outflow velocities were available, the faster value was used as the LAAFV in the statistical analysis. When either of the two was available, the value was used as the LAAFV based on the strong correlation between the outflow and inflow velocities (*r* = 0.88, *P* < 0.001).

### Treatment and follow-up

Treatment was made at the discretion of the attending physician, following practical guidelines.^6–8^ Clinical and thrombus follow-up was performed according to each centre’s standard of care, and data regarding the LA thrombi were retrospectively collected from the medical records. Given that the LAT trial aimed to investigate the clinical outcomes in patients with AF and silent LA thrombi, the details of the anticoagulation therapy were examined only twice at enrolment and the final follow-up. The follow-up period was set to 1 year after the detection of LA thrombi.

### Endpoints

The primary endpoint was LA thrombus resolution confirmed by TEE or MDCT. Patients whose LA thrombi were completely resolved without thromboembolism and surgical removal during the study period were classified as the successful resolution group. Conversely, (i) patients who experienced thromboembolism, (ii) those who underwent surgical removal of the LA thrombi, or (iii) those whose LA thrombi were not completely resolved even at the final TEE or MDCT follow-up were classified as failed resolution group.

The clinical endpoints were ischaemic stroke or systemic embolism, major bleeding, all-cause death, and a composite of the three events. Ischaemic stroke was defined as an atherothrombotic, cardioembolic, or lacunar infarction with a new focal neurologic deficit of vascular origin lasting for >24 h.^17^ Systemic embolism was defined as an acute vascular occlusion of an extremity or organ. Major bleeding was defined as intracranial haemorrhage, bleeding requiring surgery or transfusion, or a decrease in haemoglobin levels of ≥4 g/dL.

### Statistical analysis

Continuous variables are expressed as the mean ± standard deviation for normally distributed variables or as the median [interquartile range (IQR)] for non-normally distributed variables and were compared using Student’s *t*-test or Mann–Whitney *U* test, respectively. Categorical variables are presented as numbers (percentages) and were compared using the χ^2^ or Fisher’s exact test if the expected frequency was <5.

First, the patient’s baseline characteristics, OAC therapies, and TEE findings were compared between the successful and failed LA thrombus resolution groups. To examine the quality of OAC therapy, the prothrombin time-international normalized ratio (PT-INR) and time in therapeutic range (TTR) were assessed in patients prescribed vitamin K antagonist (VKA). The TTR was calculated using the Rosendaal method,^18^ assuming a linear progression of change in PT-INR between patient visits. The target range was 2.0–3.0 in this study. Second, the associations between the cardiac functional indices [echo parameters and plasma brain natriuretic peptide (BNP) levels] and LAAFV were examined using linear regression analysis. Third, receiver operating characteristic (ROC) curves were generated to determine the discriminatory ability of LAAFV as a predictor of LA thrombus resolution. The area under the ROC curve (AUC) values with 95% confidence intervals (CIs), along with the sensitivity and specificity of the LAAFV, was calculated. The best cut-off value was defined as the point that combined the highest sensitivity and specificity. Additionally, a logistic regression model was employed to assess whether the LAAFV had an incremental value for successful thrombus resolution. Fourth, a logistic regression analysis was performed to identify factors that affect LA thrombus resolution. The odds ratios (ORs) and 95% CIs were calculated. Because of the limited number of events (*n* = 39), variables significant in the univariate analysis were only included in the multivariate analysis. The backward elimination method was used in the analysis. The significance level was 0.05 for keeping a variable in the model and 0.1 for removing a variable from the model. Finally, the clinical endpoints were compared in patients with reduced (below the cut-off) and preserved (above the cut-off) LAAFV. The Kaplan–Meier curves and the log-rank test were used in the analysis. Statistical analysis was performed using JMP 13 (SAS Institute, Cary, NC, USA) and Medcalc (version 22) software. *P* values of *<*0.05 were considered significant.

## Results

The study flow chart is shown in *Figure 1*. Four out of eight patients with thromboembolism and four out of five patients who underwent surgical removal did not undergo repeat TEE or MDCT. Thus, 161 patients underwent repeat TEE (*n* = 125), MDCT (*n* = 9), or both (*n* = 27). The median number of repeat examinations was 1 (IQR, 1–1), and the median follow-up interval was 76 (34–138) days after thrombus detection (see Supplementary material online, *Figure S2*). As a result, LA thrombi were confirmed to resolve in 130 (76.9%) patients in the final TEE or MDCT. Then, 130 patients were allocated to the successful resolution group and 39 to the failed resolution group (*Figure 1*).

### Baseline characteristics

The baseline patient characteristics are described in *Table 1*. Patients with failed thrombus resolution had a more unfavourable background than those with successful thrombus resolution (e.g. lower body mass index, and higher CHADS_2_ and CHA_2_DS_2_-Vasc scores). The prevalence of heart failure and a history of stroke or transient ischaemic attack (TIA) were significantly higher in patients with failed thrombus resolution than in those with successful thrombus resolution. The laboratory data revealed lower haemoglobin levels and higher C-reactive protein (CRP) levels in patients with failed thrombus resolution than in those with successful thrombus resolution. Although there was no significant difference in the echocardiographic data, the left atrium tended to be larger in the failed resolution group. No patient had significant mitral stenosis in this study population.

**Table 1:** Baseline patient characteristics

	ALL (*n* = 169)	Successful resolution (*n* = 130)	Failed resolution (*n* = 39)	*P* value
Age, years	68 ± 10	67 ± 10	70 ± 9	0.14
Female, *n* (%)	44 (26)	31 (24)	13 (33)	0.24
Body mass index, kg/m^2^	25.0 ± 3.9	25.4 ± 3.8	23.7 ± 3.7	0.016
CHADS_2_ score	2 [1, 3]	2 [1, 3]	2 [1, 4]	0.035
CHA_2_DS_2_-Vasc score	3 [2, 4]	3 [2, 4]	3 [3, 5]	0.021
**AF type**				
Paroxysmal AF	61 (36)	51 (39)	10 (25)	0.13
Persistent AF	66 (39)	51 (39)	15 (39)	
Long-standing persistent AF	42 (25)	28 (22)	14 (36)	
**Coexisting disease**				
Heart failure	83 (49)	57 (44)	26 (67)	0.013
Ischaemic heart disease	23 (14)	17 (13)	6 (15)	0.71
Prior heart surgery	15 (8.9)	9 (7.0)	6 (15)	0.11
Hypertension	113 (67)	90 (69)	23 (59)	0.23
Diabetes mellitus	59 (35)	47 (36)	12 (31)	0.54
History of stroke or TIA	36 (21)	21 (16)	15 (39)	0.003
Malignancy	18 (11)	15 (12)	3 (7.7)	0.49
**Laboratory data**				
Serum haemoglobin level, g/dL	14.2 [12.4, 15.4]	14.2 [12.9, 15.5]	13.4 [11.5, 15.0]	0.030
Serum creatinine level, mg/dL	0.93 [0.80, 1.15]	0.92 [0.80, 1.15]	1.00 [0.83, 1.18]	0.40
High-sensitive CRP level, mg/dL	0.18 [0.07, 0.49]	0.16 [0.06, 0.40]	0.30 [0.11, 1.01]	0.017
Plasma BNP level, pg/mL	235 [112, 420]	227 [97, 425]	258 [160, 375]	0.33
D-dimer, *µ*g/mL	0.57 [0.30, 1.44]	0.51 [0.28, 1.16]	1.40 [0.43, 2.58]	0.078
**TTE measurement**				
LA diameter, mm	46 [41, 51]	46 [41, 50]	48 [42, 54]	0.094
LV diastolic diameter, mm	49 [44, 54]	49 [44, 55]	49 [44, 54]	0.94
LV systolic diameter, mm	33 [28, 42]	33 [28, 42]	31 [29, 41]	0.87
LV ejection fraction, %	60 [41, 67]	61 [40, 67]	58 [41, 67]	0.81
TRPG, mmHg	23 [19, 30]	24 [19, 29]	23 [20, 35]	0.40
Moderate-to-severe or severe MR, *n* (%)	13 (7.7)	8 (6.2)	5 (13)	0.17
**Medications, *n* (%)**				
Beta blocker	92 (54)	69 (53)	23 (59)	0.52
ACE-I or ARB	72 (43)	53 (41)	19 (47)	0.38
Antiarrhythmic drugs	51 (31)	40 (32)	11 (28)	0.67
Antiplatelet	36 (21)	26 (20)	10 (26)	0.45

ACE-I, angiotensin converting enzyme inhibitor; AF, atrial fibrillation; ARB, angiotensin II receptor blocker; BNP, brain natriuretic peptide; CRP, C-reactive protein; LA, left atrial; LV, left ventricular; MR, mitral regurgitation; NYHA, New York Heart Association; PT-INR, prothrombin time–international normalized ratio; TIA, transient ischaemic attack; TRPG, tricuspid regurgitation pressure gradient; TTE, transthoracic echocardiography.

### Oral anticoagulant prescription

**Figure 2:**
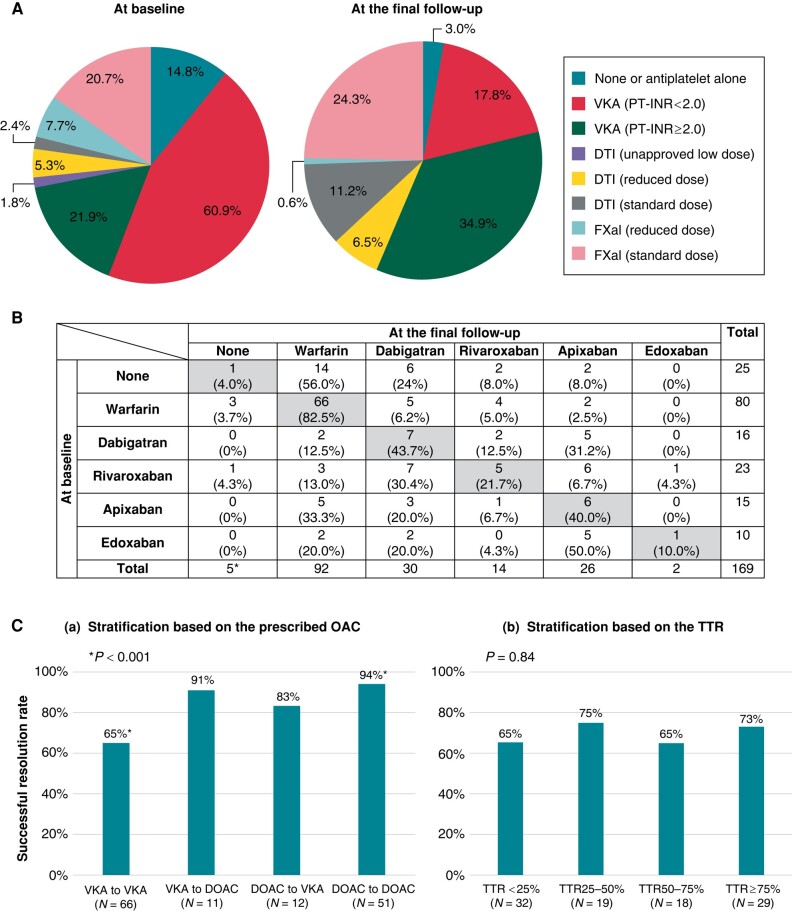
Oral anticoagulants at baseline and final follow-up. (*A*) Detailed percentages of oral anticoagulants at baseline and the final follow-up. (*B*) The table displays how to change the oral anticoagulants from baseline to the final follow-up. The number of patients prescribed each oral anticoagulant is described in each column. The vertical and horizontal columns represent the prescribed oral anticoagulants at baseline and the final follow-up, respectively. The shaded column indicates the prescription of the same oral anticoagulants at baseline and at the final follow-up. The percentages indicate the percentage of drugs used at baseline that were prescribed at the final follow-up. (*C*) Successful resolution rate stratified by (*a*) the prescribed oral anticoagulant at baseline and at the end of follow-up and (*b*) the time in therapeutic range in patients who took vitamin K antagonist during the study period. *Of the five patients without oral anticoagulant at the final follow-up, three patients died, and two patients survived. One patient died of heart failure, one died of cardioembolic stroke, and another died of acute limb ischaemia. They were in critical condition and may not have been able to undergo oral anticoagulant. Conversely, all two survivors underwent surgical LA appendage removal. Thus, oral anticoagulants therapy was no longer considered necessary. DOAC, direct oral anticoagulant; DTI, direct thrombin inhibitor; PT-INR, prothrombin time-international normalization ratio.

*Table 2* shows the details of the OAC therapy. The OAC prescription rate increased from 85.2% at baseline to 97.0% at the final follow-up (*P* = 0.002). A VKA was administered to more than half of the patients. Patients with failed resolution were more likely to receive a VKA both at baseline and at the final follow-up (*P* < 0.001). The proportion of patients with PT-INR of ≥2.0 increased from 21.9% to 34.9% at the final follow-up, and the median TTR during the study period was 50.0% (IQR, 21.3–77.1). The prescription rates of standard doses of direct thrombin inhibitors and factor Xa inhibitors increased from 2.4% to 11.2% and from 20.7% to 24.3%, respectively (*Figure 2A*).

**Table 2:** Details of the oral anticoagulants

	All (*n* = 169)	Successful resolution (*n* = 130)	Failed resolution (*n* = 39)	*P* value
**OAC at baseline, *n* (%)**				
None	25 (15)	18 (14)	7 (18)	<0.001
VKA	80 (47)	52 (40)	28 (72)	
DOAC	64 (38)	60 (46)	4 (10)	
Dabigatran [300 mg/220 mg/150 mg/110 mg] ^a^	− 16 (9.4) [4/9/2/1]	− 15 (12) [4/8/2/1]	− 1 (2.6) [0/1/0/0]	0.55
Rivaroxaban [15 mg/10 mg] ^b^	− 23 (14) [18/5]	− 22 (17) [16/5]	− 1 (2.6) [2/0]	
Apixaban [10 mg/5 mg]	− 15 (8.9) [11/4]	− 13 (10) [10/3]	− 2 (5.1) [1/1]	
Edoxaban [60 mg/30 mg]	− 10 (5.9) [6/4]	− 10 (7.7) [6/4]	− 0 (0) [0/0]	
**OAC at the final follow-up, *n* (%)**				
None	5 (3.0)	1 (0.8)	4 (10)	<0.001
VKA	92 (54)	64 (49)	28 (72)	
DOAC	72 (43)	64 (49)	7 (18)	
Dabigatran [300 mg/220 mg]	− 30 (18) [19/11]	− 26 (20) [17/9]	− 4 (10) [2/2]	0.18
Rivaroxaban [15 mg/10 mg] ^b^	− 14 (8.2) [13/1]	− 14 (11) [13/1]	− 0 (0) [0/0]	
Apixaban [10 mg/5 mg]	− 26 (15) [11/4]	− 24 (18) [10/0]	− 2 (5.1) [2/0]	
Edoxaban [60 mg/30 mg]	− 2 (1.2) [2/0]	− 1 (0.7) [1/0]	− 1 (2.6) [1/0]	

The number of patients (percentage) prescribed each OAC is described in each column. The number of patients prescribed each DOAC dosage is described within square brackets.

DOAC, direct oral anticoagulant; OAC, oral anticoagulant; VKA, vitamin K antagonist.

^a^Dabigatran 150 and 110 mg/day are unapproved reduced doses.

^b^Rivaroxaban doses are different in Japan and Western countries: 15 and 10 mg/day are approved standard and reduced doses, respectively.

*Figure 2B* presents the evolution of OAC prescriptions. Patients prescribed VKAs were more likely to continue treatment with VKAs even after the identification of LA thrombi, whereas patients with DOACs were more likely to switch to other OACs. Although the casual relationship remains unknown, patients who continued VKAs had lower successful resolution rates than those who continued DOACs [*Figure 2C* (a)]. Among patients who took VKA during the study period, the successful thrombi resolution rate did not differ across TTR in this study [*Figure 2C* (b)].

### Transoesophageal echocardiography findings and left atrial appendage flow velocity

**Figure 3:**
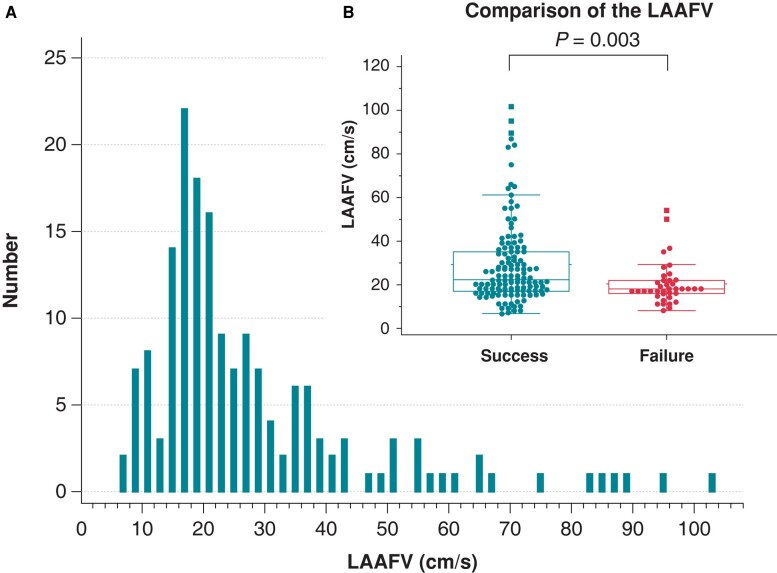
Distribution of left atrial appendage peak flow velocity. (*A*) The histograms of the left atrial appendage peak flow velocity at baseline. (*B*) Box-and-whisker and scatter plots of left atrial appendage peak flow velocity. Left atrial appendage peak flow velocity was larger in patients with successful thrombus resolution than in those with failed resolution (*P* = 0.003 by Mann–Whitney *U* test). LAAFV, left atrial appendage peak flow velocity.

The TEE findings are described in *Table 3*. Most thrombi were single, non-pedunculated, non-mobile, and located in the LAA, with a median diameter of 12 mm × 8 mm. Patients with failed thrombus resolution had larger thrombi than those with successful resolution. The distribution of LAAFV is described in *Figure 3A* and the median LAAFV was 21.0 (IQR, 17.0–32.2) cm/s. Patients with failed thrombus resolution had lower LAAFV than those with successful thrombus resolution (18.0 [15.8–22.0] vs. 22.2 [17.0–35.0] cm/s, *P* = 0.003) (*Figure 3B*).

**Table 3:** Findings of the initial transoesophageal echocardiography

	ALL (*n* = 169)	Successful resolution (*n* = 130)	Failed resolution (*n* = 39)	*P* value
**Rhythm at examination**				
Atrial fibrillation	132 (78)	103 (79)	29 (74)	0.29
Sinus	14 (8.3)	12 (9.2)	2 (5.1)	
Unknown	23 (14)	15 (12)	8 (21)	
**Heart rate, bpm**	92 [80, 117]	92 [80, 120]	94 [80, 115]	0.60
**Thrombus characteristics**				
**Number of thrombi**				
1	161 (95)	126 (96)	36 (92)	0.17
≥ 2	8 (4.7)	5 (3.8)	3 (7.7)	
**Location**				
LAA	163 (96.4)	126 (97)	35 (90)	0.084
Left atrium outside the LAA	6 (3.6)	4 (3.1)	4 (10)	
**Morphological classification**				
Pedunculated thrombi	4 (2.4)	2 (1.5)	2 (5.1)	0.20
Mobile thrombi	28 (17)	25 (19)	3 (7.7)	0.090
**Thrombus size**				
Maximum length, mm	12.0 [8.0, 17.0]	11.0 [8.0, 15.5]	16.5 [12.0, 24.4]	0.005
Maximum width, mm	8.0 [5.5, 11.4]	8.0 [5.2, 11.0]	13.0 [9.5, 22.3]	<0.001
**Other findings**				
Spontaneous echo contrast	124 (73)	97 (75)	27 (69)	0.51
Patent foramen ovale	12 (7.1)	9 (6.9)	3 (7.7)	0.87

bpm, beats per minute; LAA, left atrial appendage; LAAFV, left atrial appendage peak flow velocity.

**Figure 4:**
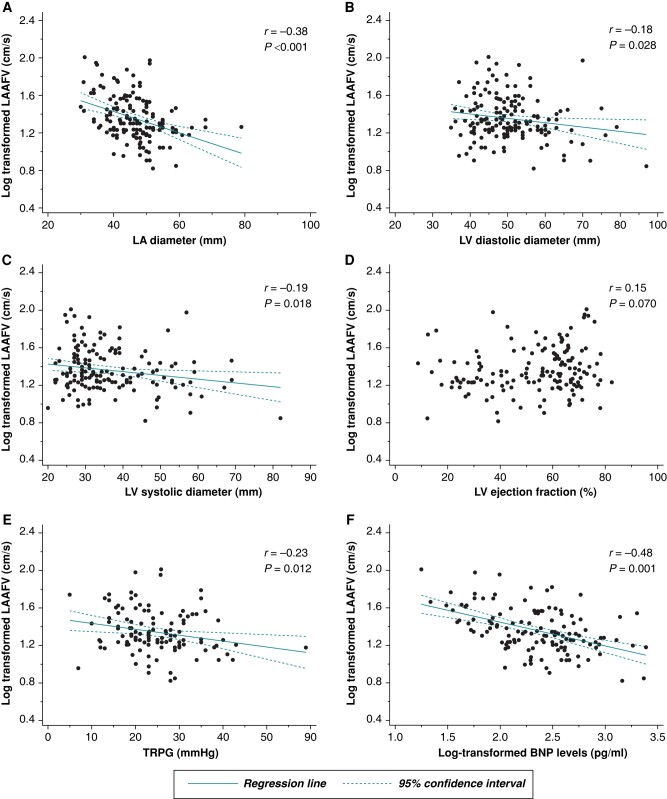
The scatter plot shows an association between the log-transformed left atrial appendage peak flow velocity and left atrial diameter (*A*), left ventricular diastolic diameter (*B*), left ventricular systolic diameter (*C*), left ventricular ejection fraction (*D*), tricuspid regurgitation pressure gradient (*E*), and log-transformed brain natriuretic peptide levels (*F*). BNP, brain natriuretic peptide; LA, left atrial; LAAFV, left atrial appendage peak flow velocity; LV, left ventricular; TRPG, tricuspid regurgitation pressure gradient.

The LAAFV exhibited weak inverse correlations with chamber size, including the baseline LA diameter (*r* = −0.16), left ventricular (LV) diastolic diameter (*r* = −0.18), and LV systolic diameter (*r* = −0.19) (all *P* < 0.050). However, it did not correlate with the LV ejection fraction (LVEF). The tricuspid annulus pressure gradient (*r* = −0.23) and plasma BNP levels (*r* = −0.18) also correlated with LAAFV (*Figure 4*).

### The discrimination ability of left atrial appendage flow velocity in successful thrombus resolution

**Figure 5:**
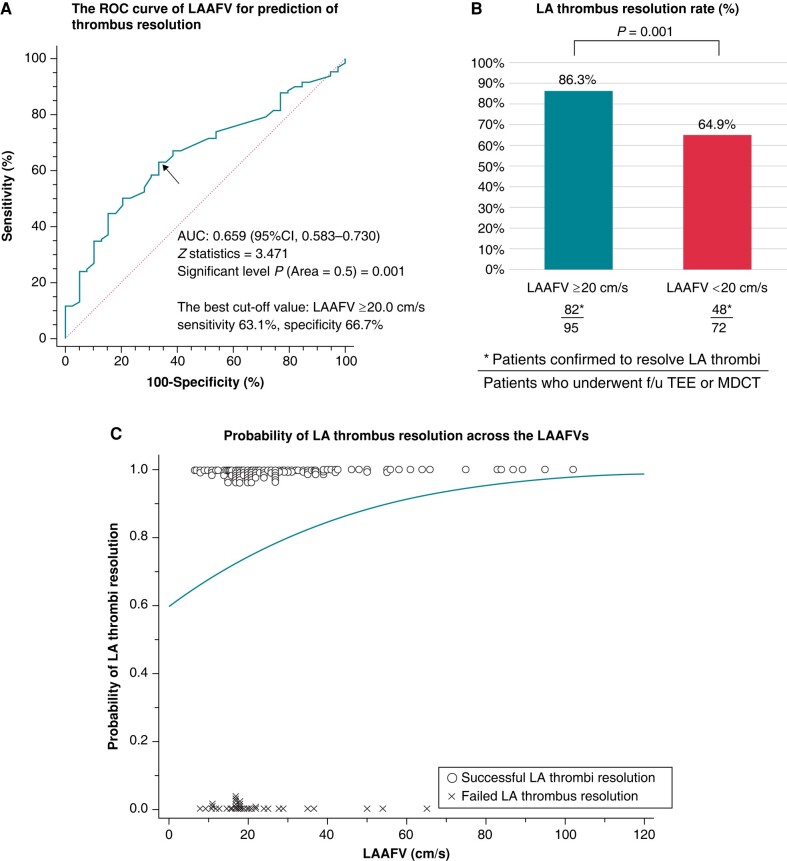
Analysis of left atrial appendage peak flow velocity and left atrial thrombus resolution. (*A*) receiver operating characteristic curve analysis revealed significant value of left atrial appendage peak flow velocity for predicting thrombus resolution. (*B*) Percentage of patients confirming left atrial thrombus resolution was compared between patients with left atrial appendage peak flow velocity ≥ 20.0 cm/s and those with left atrial appendage peak flow velocity < 20.0 cm/s. (*C*) Predicted probability curve for left atrial thrombus resolution across varying left atrial appendage peak flow velocities. Actual data points were plotted to provide a visual comparison between the predicted and observed outcomes. The incremental value of left atrial appendage peak flow velocity for predicting left atrial thrombus resolution is suggested. LA, left atrial; LAAFV, left atrial appendage peak flow velocity; ROC, receiver operating characteristic curve.

The ROC curve analysis revealed that the LAAFV had a moderate discriminatory ability for predicting LA thrombus resolution (AUC = 0.659; 95% CI, 0.583–0.730; *P* = 0.001) (*Figure 5A*). The best cut-off value of the LAAFV for predicting thrombus resolution was 20.0 cm/s, with a sensitivity of 62.3% and specificity of 64.1%. Patients with LAAFV of ≥20.0 cm/s had a better patient background and a higher rate of confirmed LA thrombus resolution than those with LAAFV of <20.0 cm/s (see Supplemental material online, *Table S3*) (*Figure 5B*). The logistic regression curve suggested an incremental value of the LAAFV (*Figure 5C*).

### Predictors of failed thrombus resolution

Logistic regression analysis was performed to identify the significant predictors of failed LA thrombus resolution. Univariate analysis revealed that body mass index, long-standing persistent AF, history of heart failure, prior stroke or TIA, serum haemoglobin levels, continued use of VKAs, continued use of DOACs, thrombi size, and baseline LAAFV were significantly associated with failed LA thrombus resolution (*Table 4*). Multivariate analysis with the backward elimination method revealed that the LAAFV (OR, 0.68; 95% CI, 0.47–0.99; *P* = 0.045) was a significant predictor of failed LA thrombus resolution. The same results were obtained when evaluating LAAFV cut-off (<20 cm/s) (OR, 2.72; 95% CI, 1.22–6.09, *P* = 0.015) (*Table 3*).

**Table 4:** Logistic regression analysis to predict failed thrombus resolution

	Univariate		Multivariate			
			Model I		Model II	
	HR (95% CI)	*P* value	HR (95% CI)	*P* value	HR (95% CI)	*P* value
Age (per 10-year increase)	1.34 (0.91–1.98)	0.14				
Female	1.60 (0.73–3.48)	0.24				
Body mass index	0.88 (0.79–0.98)	0.019	NA		NA	
Paroxysmal AF	1.00 (reference)	—	NA		NA	
Persistent AF	1.50 (0.62–3.65)	0.37	NA		NA	
Long-standing persistent AF	2.55 (1.00–6.48)	0.049	NA		NA	
Heart failure	2.56 (1.21–5.43)	<0.001	NA		NA	
Prior heart surgery	2.42 (0.80–7.30)	0.12				
Hypertension	0.64 (0.31–1.34)	0.23				
Diabetes mellitus	0.78 (0.36–1.69)	0.54				
History of stroke or TIA	3.24 (1.46–7.19)	0.004	2.86 (1.16–7.03)	0.022	2.62 (1.10–6.25)	0.030
Malignancy	0.63 (0.17–2.31)	0.48				
Serum haemoglobin levels	0.82 (0.69–0.98)	0.026	0.84 (0.69–1.01)	0.067	NA	
Baseline BNP levels (per log increase)	1.80 (0.67–4.86)	0.25				
High-sensitive CRP levels	1.01 (0.93–1.09)	0.86				
Left atrial diameter (per 10 mm increase)	1.38 (0.87–2.21)	0.17				
Left ventricular ejection fraction (per 10% increase)	1.05 (0.84–1.30)	0.67				
Antiplatelet drug use at baseline	1.11 (0.75–1.64)	0.61				
Continued use of VKA	2.91 (1.39–6.07)	0.004	NA		NA	
Continued use of DOAC	0.09 (0.02–0.39)	0.001	0.10 (0.02–0.45)	0.003	0.10 (0.02–0.43)	0.002
Switch from VKA to DOAC	0.73 (0.15–3.51)	0.69				
Switch from DOAC to VKA	0.65 (0.14–3.09)	0.59				
TTR during the study period (per 10% increase)	0.95 (0.83–1.09)	0.50				
Maximum length (per 10 mm increase)	1.05 (1.01–1.09)	0.023	NA		NA	
Maximum width (per 10 mm increase)	2.00 (1.09–3.65)	0.024	NA		NA	
LAAFV (per 10 cm increase)	0.62 (0.44–0.89)	0.009	0.68 (0.47–0.99)	0.045		
LAAFV <20 cm/s (best cut-off)	3.42 (1.61–7.27)	0.001			2.72 (1.22–6.09)	0.015

Multivariate analysis was performed using the backward elimination method. The significance level was 0.05 for keeping a variable in the model and 0.1 for removing it from the model. Variables that were considered for inclusion but were not entered into the model were described as NA. In models I and II, LAAFV was assessed using continuous and binary variables based on its cut-off value, respectively.

AF, atrial fibrillation; BNP, brain natriuretic peptide; CI, confidence interval; DOAC, direct oral anticoagulant; HR, hazard ratio; LAAFV, left atrial appendage peak flow velocity; NA, not adopted; VKA, vitamin K antagonist; TTR, time in therapeutic range.

### Impact of reduced left atrial appendage flow velocity on clinical outcomes

**Figure 6:**
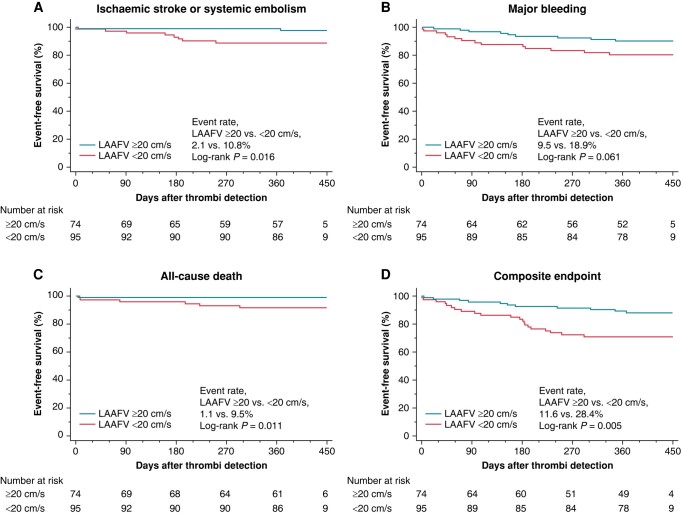
Clinical outcomes. Kaplan–Meier survival curves at the endpoint of (*A*) ischaemic stroke and systemic embolism, (*B*) major bleeding, (*C*) all-cause deaths, and (*D*) composite of the three events. The event rate was compared using the log-rank test between patients with left atrial appendage peak flow velocity ≥ 20.0 cm/s and those with left atrial appendage peak flow velocity < 20.0 cm/s. LAAFV, left atrial appendage peak flow velocity.

During the median follow-up of 394 days (IQR, 373–421) after the thrombus detection, ischaemic stroke or systemic embolism, major bleeding, all-cause death, and the composite of the three events were observed in 10 (5.9%), 23 (13.6%), 8 (4.7%), and 32 (18.9%) patients, respectively. Ischaemic stroke or systemic embolism included one atherothrombotic stroke, four cardioembolic strokes, two lancer strokes, and three systemic embolisms. The incidence of ischaemic stroke or systemic embolism was significantly higher in patients with LAAFV of < 20.0 cm/s than in those with LAAFV of ≥ 20.0 cm/s (10.8% vs. 2.1%, log-rank *P* = 0.016) (*Figure 6A*). Contrarily, there was no difference in the incidence of major bleeding (18.9% vs. 9.5%, log-rank *P* = 0.061) (*Figure 6B*). All eight patients who died during the study period (cardiovascular death, *n* = 7; non-cardiovascular death, *n* = 1) (see Supplementary material online, *Table S4*) had reduced LAAFV (9.5% vs. 1.1%, log-rank *P* = 0.011) (*Figure 6C*). The composite events were significantly higher in patients with reduced LAAFV than in those with preserved LAAFV (28.4 vs. 11.6%, log-rank *P* = 0.005) (*Figure 6D*).

## Discussion

### Major findings

The LAT study is one of the largest registries of AF patients with silent LA thrombi.^13,14^ The present study examined whether the LAAFV was associated with the success or failure of LA thrombus resolution. The principal findings were as follows: (i) LA thrombi were successfully resolved without thromboembolism or surgical removal in most patients. However, cases without successful resolutions were not rare. (ii) Patients with successful thrombus resolution had a higher baseline LAAFV than those without thrombus resolution. (iii) Reduced LAAFV (< 20.0 cm/s) was a significant predictor of failed LA thrombus resolution. Thus, assessing the LAAFV may help predict whether the existing LA thrombi are successfully resolved or not. (iv) Reduced LAAFV was also associated with adverse cardiovascular outcomes. Careful follow-up may be required in patients with reduced LAAFV.

### Resolution rate of the left atrial appendage thrombi

Data regarding the resolution of LAA thrombi are relatively sparse. Still, several retrospective studies have shown that OAC treatment has successfully resolved LA thrombi in 60–90% of patients with similar efficacy between VKAs and DOACs.^6,7,19–26^ The wide range of resolution rates is attributed to the differences in patient background (e.g. paroxysmal or persistent AF; with or without the inclusion of valvular AF; and with or without coexisting heart failure), anticoagulation strategy or treatment duration (e.g. previously anticoagulated or newly anticoagulated; 4 weeks or 12 months), and characteristics of the LA thrombi (e.g. small or large; old or newly generated). In this LAT study, LA thrombus resolution was successfully confirmed in 76.9% of patients with AF and silent LA thrombi during the 1-year follow-up. Although a few patients did not receive OACs (3.0% at the final follow-up), the resolution rate was comparable with that of previous studies.

Notably, the resolution rate was significantly lower in patients who received VKAs both at baseline and at the end of follow-up. This may reflect the difficulty of controlling PT-INR in patients with existing LA thrombi. The median TTR was 50%, suggesting a suboptimal control in the LAT study. However, given the retrospective nature of this study, the results may merely reflect that patients who were refractory to thrombolysis often received VKAs because the dose of VKAs can be modified at the physician’s discretion. Furthermore, TTR was not predictive of successful thrombi resolution in this study. Rapid thrombus resolution in most patients would partly contribute to this result. The upper threshold of PT-INR was another cause of this, as the PT-INR was controlled at >3.0 in several patients with successful thrombus resolution.

### Left atrial appendage flow velocity and cardiac functional indices

The LAAFV is a quantitative parameter of the LAA blood flow, and the median value is 50–60 cm/s in the general population.^27^ In the LAT study, each patient had varying LAAFVs with the median LAAFV of 21.0 (IQR, 17.0–32.2) cm/s. The negative correlation between the LA diameter and LAAFV indicated the importance of the LA function in LAAFV (*Figure 4*). LA structural remodelling is the most classical yet important measure associated with myocardial fibrosis and LA dysfunction.^28^ The LA reservoir strain may exhibit a stronger correlation with LAAFV, providing a potential surrogate with non-invasive transthoracic echo.^29^

On the other hand, the negative correlation of the LV chamber size, tricuspid annulus pressure gradient, and log-transformed BNP level indicated that the elevated LV filling pressure (congestion) caused blood stasis, resulting in low LAAFV. However, in this study, there was no correlation between the LVEF and LAAFV (*Figure 4*). In addition, logistic regression analysis revealed that the LAAFV, not the LVEF, was included in the multivariate model (*Table 4*). While the LVEF is one component of the fluid dynamics within the heart, the fluid dynamics within the LA and LAA may be more important in resolving existing LA thrombi. The LAAFV may be an integrated marker of the inherent LA/LAA function along with congestion, and thus, it may be more significant in thrombolysis than the mere presence of heart failure.

### Left atrial appendage flow velocity and left atrial thrombus resolution

Several studies have shown that a lower LAAFV (cut-off value varies from 11 to 50 cm/s) correlates with the formation and development of LA thrombi and ischaemic strokes.^10–12,30^ In the LAT trial, LAAFV of <20.0 cm/s was a predictor of failed LA thrombus resolution, even after consideration of the other potential confounders. However, considering the moderate discrimination ability (sensitivity, 62.3%; specificity, 64.1%), predicting LA thrombus resolution by this cut-off value alone may not be sufficient. The LAAFV is one of the three components of Virchow’s triad, and the other two components are also important for LA thrombus resolution. More importantly, the probability of successful thrombus resolution incrementally increased, and no patient with LAAFV of >54 cm/s in this study had failed thrombus resolution. In patients with relatively reduced LAAFV, combined risk stratification with a history of stroke or TIA may help us correctly stratify patients whose LA thrombi are difficult to resolve.

As demonstrated in prior studies, blood stasis increases the local fibrinogen concentration and triggers the polymerization of a fibrin network.^2,3^ Blood stasis further promotes erythrocyte adhesion to the von Willebrand Factor localized in fibrin-rich thrombi, and aggregation of erythrocytes contributes to thrombi formation.^31^ Although the coagulation cascades of thrombolysis and thrombogenesis are different, the two systems are highly regulated and interrelated with mechanisms that ensure a balanced hemostasis.^32^ The dynamic equilibrium between thrombosis and thrombolysis regulates LA thrombus formation and resolution. Although the specific role of blood flow in thrombolysis remains unclear, a reduced LAAFV may hinder thrombus resolution and promote thrombosis within the LA, especially in cases with LA thrombi.

### Left atrial appendage flow velocity and clinical outcomes

Patients with AF and silent LA thrombi are at a high risk of developing adverse cardiovascular events. In this LAT study, however, the overall incidence of unfavourable events was relatively low, probably because of the high OAC therapy rate (97% at the end of follow-up).^13^ Nonetheless, a higher rate of ischaemic stroke/systemic embolism and all-cause death was observed in patients with reduced LAAFV. The exact mechanism is still unknown and varies from patient to patient. In some cases, the residual LA thrombi may directly cause thromboembolic events and deaths, whereas in others, the underlying unfavourable backgrounds may have contributed to both reduced LAAFV and event occurrences. Atrial myopathy may be an underlying substrate associated with structural, architectural, contractile, and electrophysiological abnormalities of the left atrium and LAA, which may explain both reduced LAAFV and event occurrence.^33,34^ A minority of cases might involve thromboembolism due to LV thrombi or paradoxical embolisms via the patent foramen ovale. In contrast, the incidence of major bleeding did not significantly differ between patients with reduced and preserved LAAFV, possibly because of the limited number of major bleeding events analysed. These results demonstrated that the baseline LAAFV was a quantitative surrogate marker that could predict future adverse cardiovascular outcomes in patients with AF and silent LA thrombi.

### Clinical implications

An LA thrombus refractory to OAC therapy is a clinical conundrum. Most interventions, including cardioversion, catheter ablation, and LAA occlusion, are not recommended when LA thrombi are identified. The response to OAC therapy may be estimated by assessing the baseline LAAFV. When we identify LA thrombi in patients with reduced LAAFV (<20.0 cm/s), they are probably refractory to OAC therapy and require intensification of OAC therapy by increasing the VKA dose or switching to other OACs.^7–9^ Careful follow-up and continuation of OAC therapy may be needed in patients with reduced LAAFV because of the high risk of cardiovascular events.

### Study limitations

This study had several limitations. First, the retrospective observational design potentially included selection biases. Most patients received TEE before AF ablation and/or cardioversion, and patients with permanent AF who were not candidates for intensive therapies were not included in this study. In addition, high-risk patients, such as those with advanced heart failure or frail elderly, may not have received repeat TEE or MDCT, which may have affected the study results. Second, the follow-up method and follow-up interval were at the discretion of the attending physician. MDCT (*n* = 9) can overestimate the presence of LAA thrombi, and an arbitrary follow-up may affect the results of the analysis. Third, the treatment choice was at the discretion of the attending physician. Particularly, performing surgical removal can be arbitrary. However, based on our data (see Supplementary material online, *Table S1*), massive thrombi, major bleeding complications, and concomitant mitral regurgitation (MR) were the primary reasons for the surgical removal; this result does not deviate from standard clinical practice. Fourth, details of the anticoagulation therapy were examined only twice: at enrolment and at the endpoint (thrombus resolution or end of follow-up). Treatment adherence, discontinuation, or repetitive changes to other OACs were not recorded in the present study. Additionally, the lack of data on the prescription of CYP3A4 inhibitors or *P*-glycoprotein inhibitors made it difficult to correctly evaluate the label dosing of DOACs. Fifth, the study population was comprised entirely of patients of Asian descent, and racial characteristics may have influenced the results. Sixth, the LAA morphology and LAA orifice area,^12,35,36^ which would potentially affect the LAAFV, were not examined in this LAT study. Finally, although TEE is the gold standard for identifying LA thrombi, false-positive results would be inevitable. The use of alternative imaging, including cardiac magnetic resonance imaging, may aid in the correct diagnosis and assessment of LA and LAA fibrosis,^37^ but that was not examined in this study.

## Conclusions

In this *post hoc* analysis of the LAT study, repeat TEE or MDCT examinations revealed that residual LA thrombi were not rare in patients with AF and silent LA thrombi. Preserved LAAFV at baseline predicted successful LA thrombus resolution without thromboembolism or surgical removal. Contrarily, reduced LAAFV was associated with failed resolution of LA thrombi and adverse clinical outcomes.

## Supplementary Material

euae120_Supplementary_Data

## Data Availability

The data supporting the study findings are available from the corresponding author upon reasonable request.
