# Supplementary material for: Impact of left atrial appendage flow velocity on thrombus resolution and clinical outcomes in patients with atrial fibrillation and silent left atrial thrombi: insights from the LAT study
Source: Europace. 2024 May 1;26(5):euae120. doi: 10.1093/europace/euae120 (PMC11106584; doi:10.1093/europace/euae120)
Supplement: euae120_Supplementary_Data [file euae120_supplementary_data.zip › Supplemental Appendix OCVC Arrhythmi Investigaters.docx]

**The OCVC-Arrhythmia Investigators**

Masaharu Masuda, and Toshiaki Mano, Kansai Rosai Hospital, Amagasaki, Japan; Koichi Inoue, and Yasushi Matsumura, National Hospital Organization Osaka National Hospital, Osaka, Japan; Masato Kawasaki, Tetsuya Watanabe, and Takahisa Yamada, Osaka General Medical Center, Osaka, Japan; Miwa Miyoshi, Osaka Hospital, Japan Community Healthcare Organization Osaka, Japan; Takashi Kanda, Hitoshi Minamiguchi, Nobuhiko Makino, and Yoshiharu Higuchi, Osaka Police Hospital, Osaka, Japan; Yasuharu Matsunaga, Yasuyuki Egami, Masami Nishino, and Jun Tanouchi, Osaka Rosai Hospital, Sakai, Japan; Taiki Sato, Hirota Kida, Akihiro Sunaga, Tomoaki Nakano, Kentaro Ozu, Yohei Sotomi, Tomoharu Dohi, Katsuki Okada, Takafumi Oka, Toshihiro Takeda, Daisaku Nakatani, Shungo Hikoso, Yasushi Sakata, and Osaka University Graduate School of Medicine, Suita, Japan; Nobuaki Tanaka, Koji Tanaka, and Masato Okada, Sakurabashi Watanabe Hospital, Osaka, Japan; and Tomoko Minamisaka and Shiro Hoshida, Yao Municipal Hospital, Yao, Japan.
