# Supplementary material for: Impact of left atrial appendage flow velocity on thrombus resolution and clinical outcomes in patients with atrial fibrillation and silent left atrial thrombi: insights from the LAT study
Source: Europace. 2024 May 1;26(5):euae120. doi: 10.1093/europace/euae120 (PMC11106584; doi:10.1093/europace/euae120)
Supplement: euae120_Supplementary_Data [file euae120_supplementary_data.zip › Supplemental Figure 1 R1 submit.docx]

**Supplemental Figure 1. Representative image of the LAA thrombi and LAAFV**


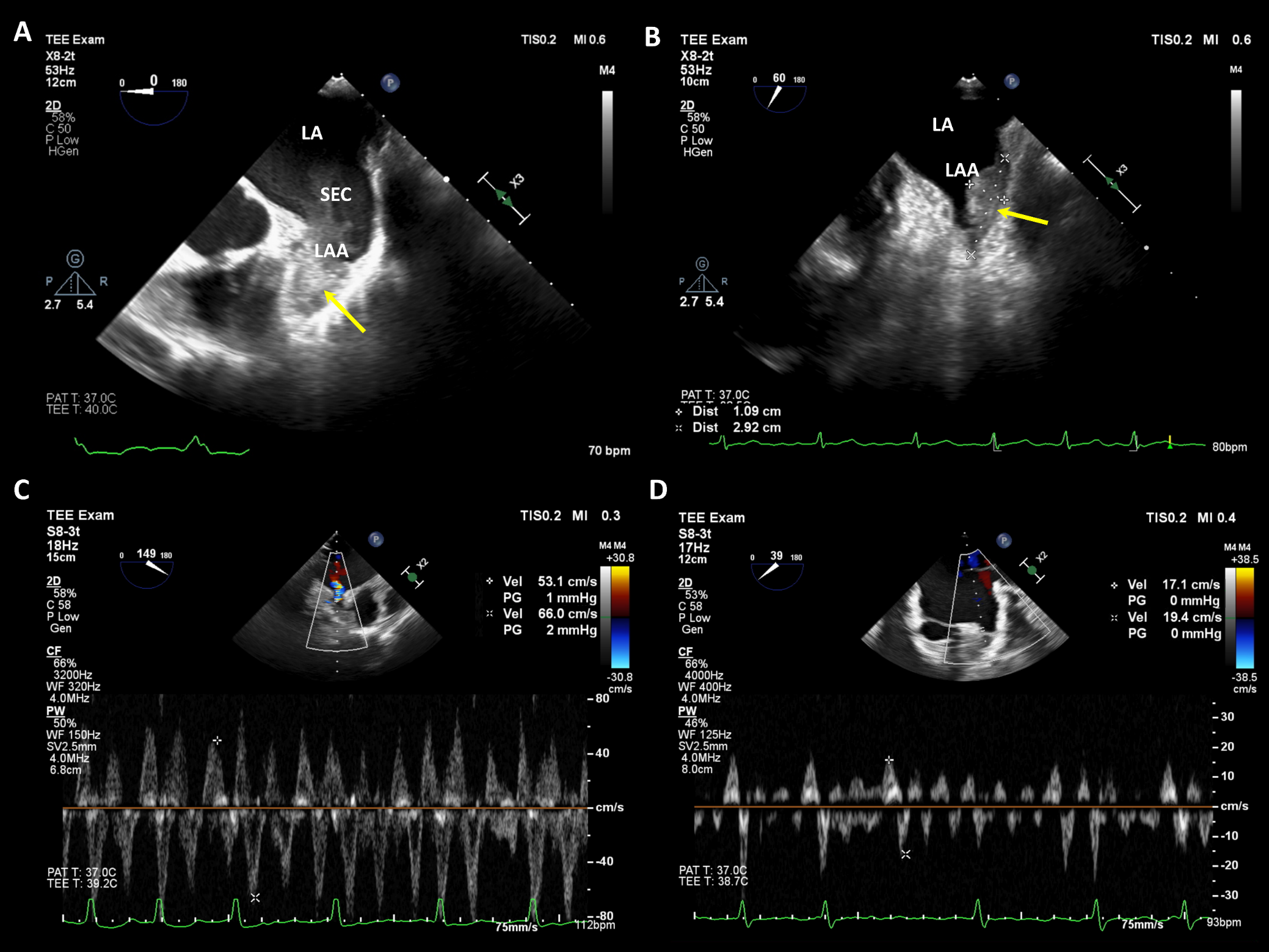


Representative image of LAA thrombi. (A) A non-mobile sand-like mural thrombus was identified in the distal portion of the LAA (arrow). (B) A mass thrombus (29.2 mm × 10.9 mm) adhering to the outer wall of the LAA was identified in the LAA body. (C) The LAAFV was obtained by pulsed-wave Doppler interrogation at the LAA entry. The measured LAA emptying and filling velocities in this case were 53.1 and 66.0 cm/s, respectively. The LAAFV was preserved in this patient. (D) The measured LAA emptying and filling velocities in this case were 17.1 and 19.4 cm/s, respectively. The LAAFV was reduced in this patient.

LA, left atrium; LAA, left atrial appendage; LAAFV, left atrial appendage peak flow velocity
