# Supplementary material for: Impact of left atrial appendage flow velocity on thrombus resolution and clinical outcomes in patients with atrial fibrillation and silent left atrial thrombi: insights from the LAT study
Source: Europace. 2024 May 1;26(5):euae120. doi: 10.1093/europace/euae120 (PMC11106584; doi:10.1093/europace/euae120)
Supplement: euae120_Supplementary_Data [file euae120_supplementary_data.zip › Supplemental Figure 2 R1 presubmit.docx]

**Supplemental Figure 2. Details of the thrombi follow-up**


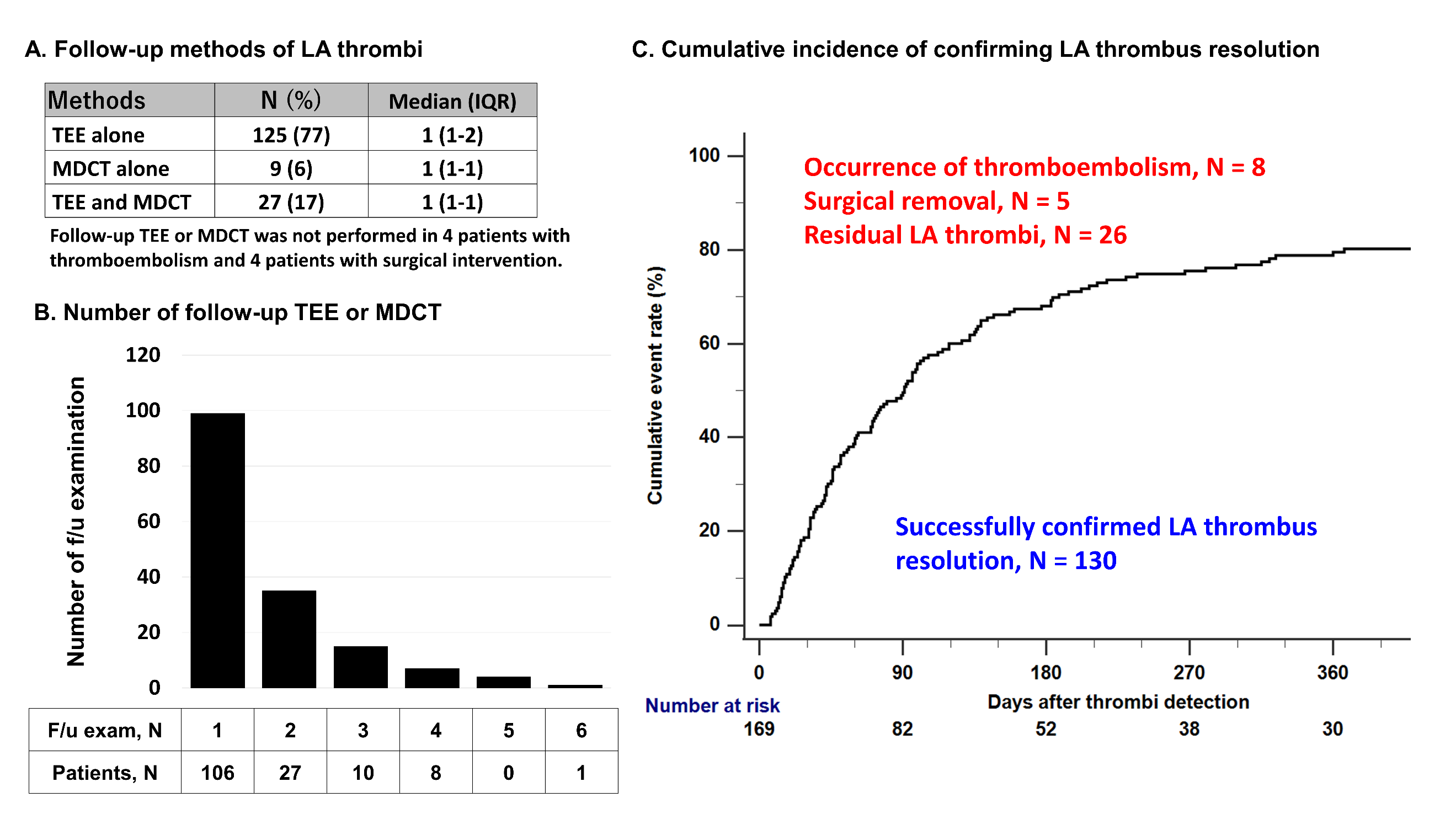


The follow-up method of the LA thrombi was at the attending physician's discretion. Thus, the follow-up tools (TEE or MDCT) (A), number (B), and intervals differed individually in this cohort.

LA, left atrial; MDCT, multidetector computed tomography; TEE, transesophageal echocardiography
