# Supplementary material for: Impact of left atrial appendage flow velocity on thrombus resolution and clinical outcomes in patients with atrial fibrillation and silent left atrial thrombi: insights from the LAT study
Source: Europace. 2024 May 1;26(5):euae120. doi: 10.1093/europace/euae120 (PMC11106584; doi:10.1093/europace/euae120)
Supplement: euae120_Supplementary_Data [file euae120_supplementary_data.zip › Supplemental Table 1 R1 presubmit.docx]

**Supplemental Table 1. Details of the eight patients who experienced thromboembolism**

| **No.** | **Days from thrombus detection to the event** | **Age, years** | **Sex** | **BMI, kg/m^2^** | **CHADS_2_-Vasc score** | **AF type** | **LAD, mm** | **LVEF, %** | **LAAFV, cm/s** | **OAC at baseline**  **(PT-INR)** | **Details of thromboembolism**  **(other information)** |
| --- | --- | --- | --- | --- | --- | --- | --- | --- | --- | --- | --- |
| **1** | 1 | 76 | M | 22.0 | 5 | PerAF | 59 | 77 | 16.0 | Warfarin  (PT-INR, 6.25) | Acute limb ischemia  (congestive heart failure with severe mitral regurgitation) |
| **2** | 4 | 78 | F | 20.2 | 7 | LS-perAF | 34 | 58 | 22.1 | Warfarin  (PT-INR, 2.38) | Cardiogenic stroke |
| **3** | 53 | 63 | M | 19.1 | 6 | LS-perAF | 51 | 28 | 12.7 | Warfarin  (PT-INR, 1.07) | Acute limb ischemia  (mechanical valve) |
| **4** | 91 | 75 | M | 20.7 | 7 | PerAF | 52 | 78 | 17.8 | Warfarin  (PT-INR, 1.18) | Cardiogenic stroke |
| **5** | 160 | 74 | M | 25.1 | 3 | PerAF | 47 | 41 | 14 | Warfarin  (PT-INR, 1.10) | Atherothrombotic stroke |
| **6** | 184 | 63 | M | 28.1 | 5 | LS-perAF | 51 | 60 | 14.7 | Warfarin  (PT-INR, 2.33) | Cardiogenic stroke |
| **7** | 191 | 79 | F | 24.9 | 8 | PerAF | 40 | 55 | 9.4 | Apixaban  (reduced dose) | Acute limb ischemia |
| **8** | 251 | 72 | F | 19.4 | 3 | PAF | 43 | 68 | 16.1 | None | Cardiogenic stroke |

AF, atrial fibrillation; BMI, body mass index; F, female; LAA, left atrial appendage; LAAFV, left atrial appendage peak flow velocity; LAD, left atrial diameter; LS-perAF, long-standing persistent atrial fibrillation; LVEF, left ventricular ejection fraction; M, male; OAC, oral anticoagulant; PerAF, persistent atrial fibrillation
