# Supplementary material for: Impact of left atrial appendage flow velocity on thrombus resolution and clinical outcomes in patients with atrial fibrillation and silent left atrial thrombi: insights from the LAT study
Source: Europace. 2024 May 1;26(5):euae120. doi: 10.1093/europace/euae120 (PMC11106584; doi:10.1093/europace/euae120)
Supplement: euae120_Supplementary_Data [file euae120_supplementary_data.zip › Supplemental Table 2 R1 presubmit.docx]

**Supplemental Table 2.** **Details of the five patients who underwent surgical removal of LA thrombi**

| **No.** | **Days from thrombus detection to surgery** | **Age, years** | **Sex** | **BMI, kg/m^2^** | **CHADS_2_-Vasc score** | **AF type** | **LAD, mm** | **LVEF, %** | **LAAFV, cm/s** | **OAC** | **Others** |
| --- | --- | --- | --- | --- | --- | --- | --- | --- | --- | --- | --- |
| **1** | 2 | 81 | F | 21.5 | 3 | Per AF | 51 | 56 | 8.0 | Warfarin | ・Massive thrombi (24.4 mm × 24.0 mm) within the LAA  ・Major bleeding complication (+): acute subdural hematoma |
| **2** | 3 | 72 | F | 27.8 | 5 | PAF | 49 | 41 | 15.7 | None | ・Concomitant severe MR |
| **3** | 22 | 75 | M | 23.7 | 3 | Per AF | 54 | 51 | 17.0 | Warfarin | ・Massive LA thrombi (48 mm × 45 mm) outside the LAA  ・Major bleeding complication (+): acute GI bleeding that requires endoscopic hemostasis (serum hemoglobin levels decreased from 12.7 g/dL to 7.8 g/dL) |
| **4** | 24 | 73 | F | 20.0 | 4 | LS-perAF | 54 | 66 | 11.0 | Warfarin | ・Unknown |
| **5** | 59 | 72 | M | 22.8 | 3 | LS-perAF | 68 | 26 | 18.2 | None  (Antiplatelet alone) | ・Concomitant severe MR  ・Major bleeding complication (+): acute GI bleeding requiring endoscopic hemostasis |

AF, atrial fibrillation; BMI, body mass index; F, female; LAA, left atrial appendage; LAAFV, left atrial appendage peak flow velocity; LAD, left atrial diameter; LS-perAF, long-standing persistent atrial fibrillation; LVEF, left ventricular ejection fraction; M, male; MR, mitral regurgitation; OAC, oral anticoagulant; PAF, paroxysmal AF; PerAF, persistent atrial fibrillation
