# Supplementary material for: Impact of left atrial appendage flow velocity on thrombus resolution and clinical outcomes in patients with atrial fibrillation and silent left atrial thrombi: insights from the LAT study
Source: Europace. 2024 May 1;26(5):euae120. doi: 10.1093/europace/euae120 (PMC11106584; doi:10.1093/europace/euae120)
Supplement: euae120_Supplementary_Data [file euae120_supplementary_data.zip › Supplemental Table 3 R1 presubmit.docx]

**Supplemental Table 3.** **Comparison of the baseline characteristics stratified by the LAAFV cutoff value**

|  | **LAAFV ≥ 20.0 cm/s**  **(n = 95)** | **LAAFV < 20.0 cm/s**  **(n = 74)** | **P value** |
| --- | --- | --- | --- |
| Age, years | 67 ± 9 | 69 ± 11 | 0.15 |
| Female, n (%) | 23 (24) | 21 (28) | 0.54 |
| Body mass index, kg/m^2^ | 25.5 ± 3.6 | 24.4 ± 4.0 | 0.063 |
| CHADS_2_ score | 2 (1–3) | 2 (1–3) | 0.003 |
| CHA_2_DS_2_-Vasc score | 3 (1–4) | 4 (2–5) | 0.003 |
| Paroxysmal AF | 45 (47) | 16 (22) | <0.001 |
| Persistent AF | 35 (37) | 31 (42) |  |
| Long-standing persistent AF | 15 (15) | 27 (37) |  |
| Heart failure | 37 (39) | 46 (62) | 0.003 |
| Prior myocardial infarction | 3 (3.2) | 5 (6.8) | 0.28 |
| Prior heart surgery | 6 (6.3) | 9 (12) | 0.18 |
| Hypertension | 66 (70) | 47 (64) | 0.42 |
| Diabetes mellitus | 35 (37) | 24 (32) | 0.55 |
| History of stroke or TIA | 13 (14) | 22 (30) | 0.006 |
| Malignancy | 9 (9.5) | 9 (12) | 0.59 |
| Serum hemoglobin level, g/dl | 14.7 (12.8–15.5) | 13.6 (12.1–15.0) | 0.027 |
| Serum creatinine level, mg/dl | 0.91 (0.80–1.10) | 1.00 (0.80–1.38) | 0.19 |
| High-sensitive CRP level, mg/dl | 0.15 (0.05–0.34) | 0.24 (0.11–0.68) | 0.003 |
| Plasma BNP level, pg/ml | 172 (73–314) | 381 (196–604) | <0.001 |
| D-dimer, µg/ml | 0.54 (0.30–1.15) | 0.61 (0.31–1.65) | 0.44 |
| LA diameter, mm | 44 (39–48) | 49 (45–53) | <0.001 |
| LV ejection fraction, % | 62 (48–66) | 55 (35–67) | 0.14 |
| TRPG, mmHg | 23 (19–28) | 26 (20–34) | 0.018 |
| Moderate-to-severe or severe MR | 3 (3.2) | 10 (14) | 0.001 |
| Antiplatelet use at baseline | 20 (21) | 16 (22) | 0.93 |
| Warfarin use at baseline | 30 (41) | 50 (52) | 0.16 |
| Diuretics use at baseline | 30 (32) | 49 (66) | <0.001 |
| Maximum length, mm | 10.0 (7.7–14.0) | 16.0 (10.3, 23.8) | <0.001 |
| Maximum width, mm | 7.3 (5.0–10.0) | 11.0 (8.0, 16.8) | <0.001 |
| Spontaneous echo contrast | 63 (66) | 61 (82) | 0.019 |
| Patent foramen oval | 8 (8.4) | 4 (5.4) | 0.45 |

Continuous variables are expressed as the mean ± standard deviation for normally distributed variables or as the median (interquartile range) for non-normally distributed variables and were compared using Student’s t-test or Mann–Whitney U test, respectively. Categorical variables are presented as numbers (percentages) and were compared using the chi-squared or Fisher’s exact test, if the expected frequency was <5.

AF, atrial fibrillation; BNP, brain natriuretic peptide; CRP, C-reactive protein; DOAC, direct oral anticoagulant; LA, left atrial; LAAFV, left atrial appendage flow velocity; LV, left ventricular; MR, mitral regurgitation; TIA, transient ischemic attack; TRPG, tricuspid regurgitation pressure gradient
