# Supplementary material for: Impact of left atrial appendage flow velocity on thrombus resolution and clinical outcomes in patients with atrial fibrillation and silent left atrial thrombi: insights from the LAT study
Source: Europace. 2024 May 1;26(5):euae120. doi: 10.1093/europace/euae120 (PMC11106584; doi:10.1093/europace/euae120)
Supplement: euae120_Supplementary_Data [file euae120_supplementary_data.zip › Supplemental Table 4 R1 presubmit.docx]

**Supplemental Table 4. Cause of death**

|  | **All**  **(n = 169)** | **Successful resolution**  **(n = 130)** | **Failed resolution**  **(n = 39)** | **P value*** |
| --- | --- | --- | --- | --- |
| **All-cause death, N (%)** | 8 (4.7) | 4 (3.1) | 4 (10.3) | 0.084 |
| **Cardiovascular death, N (%)** | 7 (4.1) | 3 (2.3) | 4 (10.3) | 0.050 |
| - Stroke deaths | 2 (1.2) | 1 (0.8) | 1 (2.6) | - |
| - Acute myocardial infarction | 1 (0.6) | 0 (0.0) | 1 (2.6) |  |
| - Acute limb ischemia | 1 (0.6) | 0 (0.0) | 1 (2.6) |  |
| - Heart failure deaths | 2 (1.2) | 1 (0.8) | 1 (2.6) |  |
| - Unexplained sudden deaths | 1 (0.6) | 1 (0.8) | 0 (0.0) |  |
| **Non-cardiovascular death, N (%)** | 1 (3.0) | 1 (0.8) | 0 (0.0) | 1.00 |
| - Cancer deaths | 1 (3.0) | 1 (0.8) | 1. (0.0) | - |

*P values were examined using Fisher's exact test.
